# Kidney Injury Molecule 1 (KIM-1): a Multifunctional Glycoprotein and Biological Marker (Review)

**DOI:** 10.17691/stm2021.13.3.08

**Published:** 2021-06-28

**Authors:** Т.А. Karmakova, N.S. Sergeeva, К.Yu. Kanukoev, B.Ya. Alekseev, А.D. Kaprin

**Affiliations:** Leading Researcher, Department of Predicting the Effectiveness of Conservative Therapy; P. Hertsen Moscow Oncology Research Institute — Branch of the National Medical Research Radiological Centre of the Ministry of Health of the Russian Federation, 3, 2^nd^ Botkinsky Proezd, Moscow, 125284, Russia; Professor, Head of the Department of Predicting the Effectiveness of Conservative Therapy; P. Hertsen Moscow Oncology Research Institute — Branch of the National Medical Research Radiological Centre of the Ministry of Health of the Russian Federation, 3, 2^nd^ Botkinsky Proezd, Moscow, 125284, Russia; Professor, Department of Biology; Pirogov Russian National Research Medical University, 1 Ostrovitianova St., Moscow, 117997, Russia; Urologist, Department of Urology with Chemotherapy; P. Hertsen Moscow Oncology Research Institute — Branch of the National Medical Research Radiological Centre of the Ministry of Health of the Russian Federation, 3, 2^nd^ Botkinsky Proezd, Moscow, 125284, Russia; Professor, Deputy General Director for Science; National Medical Research Radiological Centre of the Ministry of Health of the Russian Federation, 4 Koroleva St., Obninsk, 249036, Russia; Professor, Academician of the Russian Academy of Sciences, General Director; National Medical Research Radiological Centre of the Ministry of Health of the Russian Federation, 4 Koroleva St., Obninsk, 249036, Russia

**Keywords:** KIM-1, HAVcr-1, TIM-1, regulation of immune reactions, acute kidney injury, chronic renal failure, heart failure, renal cell carcinoma

## Abstract

KIM-1 (kidney injury molecule 1) is a transmembrane glycoprotein also known as HAVcr-1 and TIM-1 belongs to the T-cell immunoglobulin and mucin domain family (TIM) of proteins. TIM glycoproteins are presented on the immune cells and participate in the regulation of immune reactions. KIM-1 differs from other members of its family in that it is expressed not only by immunocompetent cells but epithelial cells as well. Cellular and humoral effects mediated by KIM-1 are involved in a variety of physiological and pathophysiological processes.

Current understanding of the mechanisms determining the participation of KIM-1 in viral invasion, the immune response regulation, adaptive reactions of the kidney epithelium to acute ischemic or toxic injury, in progression of chronic renal diseases, and kidney cancer development have been presented in this review. Data of clinical researches demonstrating the association of KIM-1 with viral diseases and immune disorders have also been analyzed. Potential application of KIM-1 as urinary or serological marker in renal and cardiovascular diseases has been considered.

## Introduction

Biological markers are measurable molecular, biochemical, or structural indicators of the state of the cells, tissues, or organs which are currently used in practical medicine, preclinical, and experimental investigations owing to the development of biomedical technologies.

Not so long ago, membrane glycoprotein KIM-1, also known as HAVcr-1 and TIM-1, joined these markers.

In 1996, Kaplan et al. [1] were the first to describe mucin-like membrane glycoprotein type I homologous to the immunoglobulin family proteins which facilitated the penetration of the hepatitis A virus to the cultivated cells of the African green monkey kidney. This glycoprotein was called HAVcr-1 (hepatitis A virus cellular receptor 1). The DNA sequence homologous to *havcr-1* was also detected in the human genome [1].

In 1998, Ichimura et al. [2], studying post-ischemic reparation of renal epithelium in rats, identified *Kim-1* gene (kidney injury molecule 1), whose high expression was typical for epithelial cells of the damaged proximal renal tubules. This gene appeared to be a full homolog of *HAVcr-1* [2].

In 2001, McIntire et al. [3] found a gene cluster controlling hyperactivity of the respiratory epithelium (T-cell and airway phenotype regulator, *Tapr*) in mice resistant to the asthmatic reaction development. A group of genes included in this cluster was referred to a separate family, TIM (T-cell immunoglobulin and mucin domain family), called so due to the structural similarity of the encoded proteins. One of the genes, *Tim-1*, appeared to be a close homolog of *Kim-1* in rats and *HAVcr-1* in humans and primates [3]. Later, there were identified eight proteins, members of the TIM family (TIM-1, …, -8) in mice, six proteins (TIM-1, …, -6) in rats, three proteins TIM-1 (KIM-1), TIM-3, and TIM-4 in humans [4].

At present, *HAVcr-1* gene bears its historical name in biological databases while in publications its product preserves the name commonly accepted in the appropriate field of investigation, i.e. HAVcr-1, KIM-1, or TIM-1 (CD365).

The TIM family glycoproteins are mostly expressed by the cells of the immune system and are involved in various physiological and pathological processes associated with the regulation of immune reactions [5]. KIM-1, unlike other members of the TIM family, is presented not only on lymphocytes but in other types of cells determining diverse manifestations of its functional activity.

The latest data on physiological and pathophysiological properties of HAVcr-1/KIM-1/TIM-1 have been systematized in the present review, some aspects of using this glycoprotein as a marker in clinical investigations have also been studied.

## KIM-1 molecule structure

In the human genome, *HAVcr-1* (Gene ID: 26762) is located on the long arm of chromosome 5 at locus 5q33.3 and contains 11 exons. Variants of KIM-1 generated as a result of an alternative mRNA splicing may contain from 334 to 401 amino acids with variations of glycoprotein molecular mass from 36 to 44 kDa [6, 7]. The molecular mass of a mature (fully glycosylated) KIM-1 reaches 104 kDa [6]**.**

KIM-1 is localized on the plasma membrane forming extracellular, transmembrane, and cytoplasmic domains (see the Figure) [8]. The extracellular part of KIM-1 includes a globular domain similar to the variable fragment of immunoglobulins (IgV), mucin-like sequence, and a short peptide segment.

**Figure:**
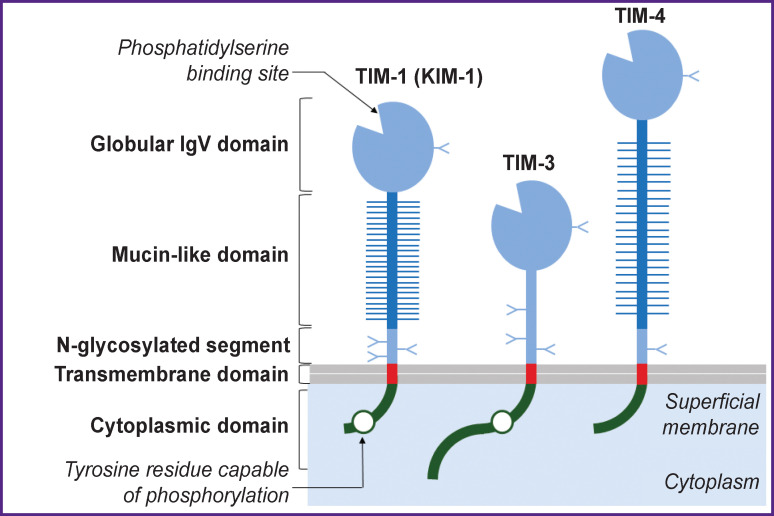
Schematic structure of the TIM family glycoproteins (based on the materials of Kuchroo et al. [8])

A key feature of the KIM-1 IgV-domain structure is the presence of a hydrophobic “pocket” (metal-ion-dependent ligand binding site, MILIBS), capable of binding a signaling phospholipid, phosphatidylserine (PS) [9]. Normally, PS is localized on the inner side of the cell plasma membrane and during apoptosis induction moves to the outer side of the membrane. It serves as a signal for macrophages and epithelial cells to absorb the dying cells [10]. It is believed that due to the ability to interact with PS, KIM-1 in the epithelial cells can perform the function of a scavenger receptor mediating *in situ* elimination of cellular debris in case of tissue damage [11]. Glycoproteins of the TIM family [12, 13], oxidized low-density lipoproteins [14], P-selectin [15], and conjugated blood bilirubin [16] are also potential ligands binding to which is mediated by IgV domain.

Highly glycosylated mucin-like domain, the most massive part of KIM-1, contains tandem repeats of amino acids and sites of O-glycosylation. No specific functional properties have been described for this domain; however, it is assumed that the organization of this molecular segment is important for the KIM-1 interaction with ligands [8, 17]. In primates, this domain is characterized by a high genetically determined polymorphism [18].

A short peptide segment which is located immediately at the membrane contains N-glycosylation sites and sequences sensitive to metalloproteinases. As a result of proteolytic cleavage, an extracellular part of KIM-1 is shed from the cell surface and a free form of KIM-1 with molecular mass of about 90 kDa is formed which can be identified in blood plasma and urine [6].

A KIM-1 cytoplasmic domain is a variable short polypeptide differentiated in the structural KIM-1 variants [6]. In renal epithelium and lymphocytes, this domain is capable of phosphorylation which determines the possibility of involving KIM-1 in intracellular signaling [6, 17].

## KIM-1 expression in tissues

In normal human tissues, expression of mRNA encoding the HAVcr-1 protein is characterized by marked organ specificity [19]. The greatest amount of *HAVcr-1* transcripts was found in the kidney tissue, less but significant — in the testicle tissue. In other tissues, specific transcripts were identified in trace quantities. Current data obtained by deep transcriptome sequencing confirm that content of *HAVcr-1* transcripts in the kidney is 10 times higher than in the majority of other organs and tissues [7]. Significant amounts of *HAVcr-1* mRNA are detected in the tissue of the colon and rectum, testes, and also in peripheral blood leukocytes [7].

Immunological studies using polyclonal antibodies to the recombinant HAVcr-1 demonstrate weak or moderate cytoplasmic staining in the cells of kidney tubule epithelium and urothelium, in the glands of the small and large intestine, epithelium of the liver bile ducts and gall bladder, bronchial epithelium, endometrium, etc. [7]. Additionally, HAVcr-1 is detected in oligodendrocytes of the human brain and myocytes of the skeletal muscle tissue. Thus, *HAVcr-1* is expressed practically in all human organs and tissues but normally the level of its expression is low.

## KIM-1 and viral infection

TIM family glycoproteins may serve as a site of entry for viral infection due to the fact that enveloped and pseudo-enveloped viruses use the ability of IgV-domain to bind PS to penetrate the cell [20]. Virus particles released from the dying cells trap the fragments of the host cell surface membrane containing PS (apoptotic mimicry) [21]. PS contained within the viral envelope interacts with TIM-1 molecules on the surface of other target cells promoting the attachment of virions and facilitating their internalization [20].

TIM-1 has been shown to mediate the penetration of hepatitis A and C viruses, human immunodeficiency virus (HIV), Ebola, Marburg and dengue viruses, Japanese encephalitis virus into the cells [20]. The effect of TIM-1 on the pathogenesis of viral diseases is confirmed by the fact that some of its polymorphisms are associated with susceptibility or, on the contrary, resistance to HIV infection [22], filoviral infection [23], viral hepatitis agents [24, 25]. It has been established in the experimental studies that “a cytokine storm”, a massive release of proinflammatory mediators, which occurs in Ebola virus infection, is induced in PS-mediated interaction of the virus with TIM-1 on the surface of T-lymphocytes. PS blockage decreases binding of the virus to the cells *in vitro* while the TIM-1-knockout mice survive when fatally infected by the virus [26].

At the same time, the role of TIM-1 in the development of, for example, HIV, is ambiguous: on the one hand, TIM-1 expression enhances virus internalization, on the other, the same TIM-1 inhibits the release of viral particles from the cell by binding and accumulating viral agglomerates at the cell surface, i.e. turns the cell to a “trap” for the virus [27].

A high polymorphism of the *HAVcr-1* gene in primates is supposed by the investigators to be the result of evolutionary divergence of the mechanisms of adaptation to viral infections and allergic reactions in mammals [18].

## KIM-1 and immune reactions

KIM-1/TIM-1, presented on lymphocytes, participates in the formation of both immunostimulating and immunosuppressive reactions. The character of immune TIM-1-mediated effects depends on the cells carrying it, interacting ligands, and their concentration as well as on the cellular microenvironment [12, 17, 28]. Realization of immune reactions in which TIM-1 is involved is influenced by a cooperative participation of other glycoproteins of the TIM family. TIM-4 in this case is a co-stimulating factor in cell interactions, while TIM-3 is an instrument of reciprocal regulation of the TIM-1-related reactions of the innate and acquired immune response [29].

TIM-1 has been established to be involved in the regulation of the T-cell immunity determining the differentiation and clonal expansion of T-helpers and corresponding polarization of the immune response in the direction of the predominance of Th2-, Th1-, or Th17-mediated effects [30]. TIM-1 activation by its agonists results in the development of the rapid proinflammatory response *in vitro* and *in vivo* [31, 32].

TIM-1 is closely related to the formation of the TCR-complex which is responsible for the recognition of antigens presented by the molecules of the major histocompatibility complex on T-lymphocytes [33]. In the naive lymphocytes, TIM-1 is mainly contained in endosomes, and under the activating stimulus, it is transported to the cell surface and concentrates in the region of the immune synapse [34]. Phosphorylation of the cytoplasmic TIM-1 domain in the TRC-complex is part of activation of PI3K/АКТ signaling pathway cascade [35]. Ligands interacting with high affinity with TIM-1, for example, PS, bind glycoprotein exposed on the superficial membrane and therefore may reduce its contribution to the T-cell activation [34].

Interacting with P-selectin, TIM-1 is able to participate in the processes of adhesion and leukocyte movement in inflammatory response [36]. Acting as a PS receptor, TIM-1 promotes phagocytosis of apoptotic cells by macrophages, which inhibits autoimmune reactions [9] and activates invariant NKT cells as well [31].

Most information on the role of TIM-1 in the regulation of immune reactions was obtained from the investigations on animals (mice). However, regulation and functions of TIM-1 in rodents and primates are likely to differ essentially [33]. So, locus *Tapr* in mice contains 8 genes. Five of them are absent in humans including *Tim-2* gene coding glycoprotein which acts as an inhibitor of TIM-1-mediated signaling. Besides, proteins of TIM family are able to bind various natural ligands [30], therefore, it is next to impossible to anticipate the integral result of these interactions in the model studies.

The role of TIM-1 in the regulation of immune reactions in a human is indirectly confirmed by the fact that genetic TIM-1 polymorphisms in some ethnic populations are associated with bronchial asthma [37–40] and systemic lupus erythematosus [41], correlate with the inflammatory reaction intensity, and the severity of community-acquired pneumonia in children [42].

The greatest interest to TIM-1 in immunological studies is connected with its role in the physiology of regulatory cells. It has been shown that the interaction of TIM-1 with the antagonist ligands is a stimulating factor for suppressor T-regulatory cells (Тreg) [43]. The reduced TIM-1 expression on the Тreg cells is noted in patients with diabetes mellitus type I [44]. TIM-1 expression is typical for one of the regulatory B cells (Breg) subpopulations producing IL-10, the main negative regulator of the inflammatory response [45, 46]. In the clinical studies, there has been revealed the correlation between a high content of TIM-1 positive (TIM-1^+^) B cells in peripheral blood and a favorable prognosis in patients with acute respiratory distress syndrome [47]. Decreased amount of TIM-1^+^ Breg cells in peripheral blood is noted in patients with progressive systemic sclerosis in comparison with healthy donors [48]. An elevated TIM-1 expression on Breg cells correlating with the disease severity is described in myasthenia gravis [49].

TIM-1^+^ Breg cells attract special attention due to their probable participation in the suppression of antitumor immune reactions. Thus, in patients with hepatocellular carcinoma, a relative content of TIM-1^+^ В cells in peripheral blood and in tumor tissue is statistically significantly higher than in healthy donors and in non-tumor liver tissue, respectively [50, 51]. Similar tendency was revealed in lung cancer [51]. The degree of infiltration of hepatic carcinoma tissue by TIM-1^+^ В cells directly correlates with the disease stage and early tumor recurrence after surgical treatment [51].

On the whole, TIM-1 today ranks among the critical immune checkpoints which may serve as targets for therapeutic action aimed, inter alia, at the activation of antitumor immune reactions [52–54].

## KIM-1 in acute kidney injury

Acute kidney injury (AKI) may be the result of shock condition and acute cardiovascular insufficiency, toxic or septic injury, urinary duct obstruction [55]. The most common AKI cause is ischemia [56] leading to the death and exfoliation of tubular epithelial cells from the basement membrane. AKI is a potentially reversible disorder since kidney tubule epithelium possesses high regeneration ability. The cells with preserved viability undergo epithelial-mesenchymal transition, proliferate, and migrate to the areas of the exposed basement membrane where they return to the differentiated epithelial phenotype [57].

It was shown in various AKI models in rats that KIM-1 expression on the apical surface of the epithelial cells of the renal proximal tubules is induced by ischemia and toxic damage [2, 58]. Iсhimura et al. [2] were the first to suppose that this phenomenon is associated with the processes of renal epithelium regeneration. Later, Zhang et al. [59] established a direct relationship between the increased KIM-1 expression in the tissue of the transplanted kidney and its functional restoration. Thus, the increase of KIM-1 production in the proximal tubular cells in AKI is likely to be an adaptive response, being not only the marker of injury but the reflection of the reparation activity [59].

Investigations on animals and cell cultures show that in AKI KIM-1 is directly involved in the processes of preservation and restoration of the structural and functional integrity of the epithelium of proximal nephron compartments [14, 60]. It is supposed that possessing the features of PS, KIM-1 may induce phagocytosis of dying cell residues maintaining thereby cleaning of the proximal tubular lumen from cellular debris and decreasing the probability of filtrate flow impairment [14]. Increase of KIM-1 expression on the surface of the proximal tubular epithelial cells is induced by the presence of albumin in the glomerular filtrate [61]. In this case, KIM-1 can capture albumin from the primary urine, carry it into the cell which supplements the main receptor mechanisms of protein reabsorption in the kidneys in severe proteinuria [61].

An elevated KIM-1 expression in the renal proximal tubular cells inhibits proliferation and activity of effector T cells and leads to the increase of Treg cell content in the kidney tissue providing local formation of immune tolerance conditions and prevents the development of autoimmune reactions [60]. Entrapment of apoptotic bodies by the renal epithelial cells induces interaction of cytoplasmic domain KIM-1 with protein p85. This stimulates autophagia and inhibits the activity of transcription factor NFkB responsible for the proinflammatory cytokine production by the cell [60, 62]. Besides, the cytoplasmic domain KIM-1 is able to influence the G protein activity, pleiotropic regulator of intracellular signaling processes, reducing the probability of cell damage in ischemia and hypoxia [63]. Increased KIM-1 production can result in the reduced intracellular content of nuclear receptor Nur77, an inductor of apoptosis, preventing the programmed cell death and enhancing their survivability in stress conditions [64]. KIM-1 expression has also been shown to promote migration and proliferation of dedifferentiated cells in the regenerating renal epithelium [65].

Proteolytic cleavage of the extracellular domain KIM-1, which occurs normally in the renal epithelial cells, determines the basic level of glycoprotein in urine. This process is enhanced many times when cells are damaged. Presence of serum albumin, production of TNF-α by immune cells, and active forms of oxygen may serve as stimuli for the enhancement of KIM-1 cleavage [66]. A free KIM-1 form has been shown to interact with integrin on the apical cell surface which may prevent aggregation of the cleaved cells and diminish the risk of tubule obstruction [67].

When renal epithelial cells die, a soluble KIM-1 together with the fluid may be entrained to the interstitium and enter therefrom the bloodstream [68]. Higher KIM-1 concentration in blood together with its increased contents in urine can also reflect injury of the kidney tubular apparatus [69].

KIM-1 possesses the properties of ideal marker of renal proximal tubule epithelium injury [70]: in the normal kidney, KIM-1 expression is determined in trace quantities; in ischemic or toxic kidney injury, activation of KIM-1 synthesis in the cells of the damaged tubules and its increased expression on the apical cell membrane is observed; shedding of KIM-1 from the cell surface results in considerable increase of its content in urine and/or in the circulating blood. According to the data of the experimental studies on animals, KIM-1 expression in the epithelial cells of the renal proximal tubules as well as its concentration in urine and blood plasma correlate with the severity of the pathological process in the kidneys [70]. Elevation of KIM-1 level in urine (uKIM-1) is a more sensitive indicator of AKI than the reduction of creatinine clearance or albuminuria. It is underlined that enhanced excretion of KIM-1 in urine is highly specific for the conditions caused by kidney injury since, due to a large molecular mass, free KIM-1 entering the blood from extrarenal sources, is not filtered through the glomerular barrier. Moreover, no other body tissue produces KIM-1 in the quantities capable of essential influence on its content in urine [70].

In 2010, FDA (US Food and Drug Administration), and EMEA (European Medicines Agency) included uKIM-1 into the number of the markers of drug nephrotoxicity for preclinical trials [71]. This was the impetus for widening the sphere of studying uKIM-1 and its content in blood plasma and serum (pKIM-1 or sKIM-1) [72]. According to the data of meta-analysis summarizing the results of clinical investigations carried out from 2008 to 2013, sensitivity and specificity of uKIM-1 as a predictor of AKI development were 81.8 and 83.8%, respectively [73]. KIM-1 as a marker of kidney injury appeared to be promising in translational research in rodents [69, 74], dogs [75], cats [76], and Danio rerio (zebrafish) [77].

Numerous publications give convincing evidence that the increased uKIM-1 level is a reliable indicator of drug therapy nephrotoxicity. It is known that application of anticancer medication (chemotherapy) can not only cause AKI but also provoke chronic renal disease [78]. Urea nitrogen concentration and creatinine clearance, which characterize nephron filtration activity, are considered to be classic indicators of drug-induced nephrotoxicity. However, administration of cisplatin, doxorubicin, or methotrexate does not result in serious impairment of the renal glomerular function but the main damage is observed in the proximal tubular epithelium which accumulates these preparations to the greatest degree [79]. According to quite a number of clinical studies [80, 81], uKIM-1 is a highly sensitive and specific marker of nephrotoxicity induced by cisplatin and is superior in this respect to other urinary markers both classic and new.

In HIV carriers receiving antiretroviral therapy, determination of uKIM-1 level is recognized advisable for diagnosing renal function disorder, predicting progression of renal insufficiency, and the related risk of lethal outcome [82]. Increase of uKIM-1 has been established in AKI caused by treatment with vancomycin [83], adefovir [84], and paracetamol overdosage [85].

Findings of several studies have shown that uKIM-1 is an early marker of AKI induced by the administration of the contrast agent to the patients who undergo coronary or peripheral angiography [86, 87], and pKIM-1 may serve as a prognostic marker of concurrent chronic kidney injury development [88].

Of special interest are data on the possibility of using uKIM-1 as a noninvasive marker for the assessment of functional state of the transplanted kidney [89]. Inevitable ischemia during organ transplantation results in the renal tissue injury to various degree which is accompanied by the increased KIM-1 expression in the epithelial cells of the renal proximal tubules. The analysis of biopsy specimens found direct correlation between KIM-1 expression in the kidney allograft tissue and fibrosis [90] and inverse correlation with tubular epithelium condition [91]. Increased uKIM-1 and sKIM-1 have been shown to accompany acute or delayed allograft rejection [92] and to occur several months earlier than the clinical signs of acute renal dysfunction [93].

There exist data indicating that uKIM-1 correlates with the AKI development in sepsis [94], and sKIM-1 elevation is a predictor of exacerbation of urinary tract infections in children [95]. In congenital ureteral stricture in children, identification of uKIM-1 in combination with urinary NGAL and RBP may be useful for the monitoring of kidney condition and in making a decision on the necessity of surgical intervention [96].

## KIM-1 in chronic kidney diseases

Chronic kidney disease (CKD) is a natural outcome of the majority of nephropathies and can be provoked by acute and recurrent kidney injury of various etiologies [97]. The main pathogenetic mechanism of progressive CKD is the condition of chronic hypoxia which occurs due to structural and functional disorders in the network of post-glomerular capillaries, excessive activity of renin-angiotensin system, compensatory increase of oxygen consumption by the renal tissue cells under the condition of deterioration in glomerular function and oxidative stress [98]. Chronic hypoxia leads to sclerotic glomerular changes and tubulointerstitial fibrosis [98]. Besides, it is supposed that in CKD, an important role in the development of these disorders is played by the acquisition of mesenchymal phenotype by the cells of the damaged proximal tubules and the release of profibrotic factors by them [99].

Hypoxia is a powerful stimulus of KIM-1 expression increase in proximal tubular cells which, in its turn, may result in the induction of chronic interstitial inflammation [89, 100]. Membrane-bound as well as free KIM-1 are considered to be involved in signaling interactions between the cells of the damaged renal proximal tubules and macrophages acting as autocrine-paracrine factor in relation to the epithelial and stromal cells [62, 66, 101]. In particular, interaction of KIM-1 with LMIR5/CD300b receptor on the resident myeloid cells has been shown to lead to the release of cytokines and chemokines which attract neutrophils to the focus of injury. This provokes enhancement of local inflammatory reactions, hypoxia, and cell damage [102]. Elevated KIM-1 expression under hypoxic conditions promotes CKD progression creating a positive feedback loop which is completed with interstitial fibrosis [103]. Thus, functional KIM-1 effects may be a connecting mechanism in the pathogenesis of acute and chronic renal disorders.

Increase of KIM-1 expression and an elevated uKIM-1 level are described in focal glomerulosclerosis, proliferative and membrane glomerulonephritis, IgA nephropathy, diabetic and hypertensive nephropathy, chronic allograft nephropathy, lupus nephritis, etc. [89]. However, the prognostic significance of uKIM-1 in СKD seems to be limited [104, 105]. Seibert et al. [106] did not find reliable link between uKIM-1 and indicators of kidney function in patients with chronic diseases and associate it with the fact that in the majority of patients enrolled in the study such diseases were caused by the preferential damage of the glomerular apparatus. At the same time, the authors note that in IgA nephropathy, membranous and lupus nephritis, uKIM-1 inversely correlates with the glomerular filtration rate, i.e. in some chronic renal inflammatory lesions, uKIM-1 may pretend to the role of a biological marker [106]. Data on the correlation of uKIM-1 with the severity of IgA nephropathy [107], tubular atrophy, and tubulointerstitial inflammation in patients with systemic lupus erythematosus [108], activity of chronic glomerulonephritis, and efficacy of treatment of this disease agree with this conclusion [109, 110].

The analysis of data from five cohort prospective studies, included patients with atherosclerosis, diabetes, chronic kidney diseases, as well as elderly people referred to the conventional group of risk, has shown that increased uKIM-1 correlates with the reduction of glomerular filtration rate, albuminuria, and the risk of chronic renal failure development which confirms close interrelation between glomerular and tubular dysfunction [111].

Findings of clinical investigations [112] demonstrate that the uKIM-1 level rises in diabetic nephropathy including patients with normal or slightly increased albumin in urine. However, uKIM-1 is likely to have no essential preference as a marker of kidney injury in diabetic patients in comparison with the traditional laboratory parameters [113].

It should be noted that the increased KIM-1 level in blood in CKD may have a greater clinical significance than its content growth in urine. Thus, in patients with diabetes mellitus type I, rise of KIM-1 levels in blood correlates with the reduction of glomerular filtration rate allowing this glycoprotein to be considered as an early marker of renal failure progression [69, 114].

## KIM-1 in cardiovascular diseases

Physiological interconnection between the activity of kidneys, heart, and vessels creates the situation when dysfunction of either of these systems aggravates disorders in the entire cardiorenal continuum [115]. Searching for new predictive markers of cardiovascular diseases showed that pKIM-1 reflects the severity of the state for patients with heart failure [116]. In elderly diabetic men, uKIM-1 growth is associated with the risk of death from cardiovascular complications irrespective of other indicators [117]. Egli et al. [118] during clinical examination of 2060 conditionally healthy people at the age of 25‒41 years have revealed that рKIM-1 levels do not correlate with the indices of renal function impairment (creatinine and cystatin C) in this population but is statistically significantly associated with the risk factors of cardiovascular diseases: high arterial pressure, blood content of low- and high-density lipoproteins as well as C-reactive protein.

Wybraniec et al. [119] have followed-up 95 patients after coronary angiography for 12 months and established that the uKIM-1 levels increase after this diagnostic procedure is an independent predictor of infarction or stroke for these patients in the remote period.

Some investigators believe that the elevated uKIM-1 levels may serve as an early diagnostic indicator of AKI in patients after cardiac surgery [73] and correlate with the length of acute period of renal disorders [120]. According to other data [121, 122], uKIM-1 is low informative to assess the development of renal complications in patients after heart surgery, but its increase is associated with a lethal outcome. A more accurate prognostic assessment may be obtained combining uKIM-1 with other markers of kidney injury, i.e. NGAL in blood plasma and IL-18 in urine [121], or cysteine C in blood serum and NGAL in urine [122]. It has been shown that in patients with heart failure or atherosclerosis, uKIM-1 in combination with cysteine C may serve as an early marker of AKI [123] and a risk factor of renal failure progression up to the terminal stage [124], and prognostic significance of uKIM-1 within the frames of such assessment exceeds other urinary markers of renal injury (NAG, NGAL, and L-FABP) [125].

## KIM-1 in renal cancer

Renal cell carcinoma (RCC) is a malignant neoplasm originated from the renal tubular epithelium. The majority of cases of primary sporadic RCC are tumors derived from the cells of the renal proximal tubules: clear cell RCC (75‒92%) and papillary RCC (4–16%) [126, 127]. Chromophobe RCC occurs not so commonly (2.4–5.0%) [126, 127], it is thought to develop from the epithelium of the distal nephron parts.

Immunohistochemical evaluations show that an elevated KIM-1 expression in clear cell carcinoma is observed in 71–100% cases, in papillary RCC in 80–91% cases [128–131]. A relatively high rate of KIM-1 expression (74%) was found in the tissues of nephroblastoma in children [132]. KIM-1 is determined extremely rarely in the cells of chromophobe RCC as well as benign oncocytomas [128, 131].

KIM-1 is supposed to play a functional role in the pathogenesis of renal carcinomas and disease progression. According to the data of *in vitro* studies, KIM-1 expression in the human umbilical vein endothelial cells (HUVEC) transfected by *HAVcr-1* increases their disintegration and sensitivity to the action of hepatocyte growth factor destroying intercellular contacts [133]. KIM-1 has been detected [134] to interact with Rho GTPase which regulates the dynamics of the assembly of cytoplasmic ZO-protein complex supporting the structure of the tight junctions. This can promote disintegration of the tumor cells and facilitate metastasizing.

TIM-1-mediated regulation of the nuclear receptor Nur77 degradation results in the inhibition of apoptosis signals in the cells of human renal carcinoma and immortalized cells of the renal epithelium [64]. This mechanism which maintains the integrity of the renal tubular epithelium in ischemic injury may contribute to the survival of the tumor cells.

IL-6 is known to be a key factor of tumor-associated inflammation, while co-expression of IL-6 and its receptor in the RCC tissue are associated with a poor prognosis in renal carcinoma patients [135]. Cuadros et al. [136] report that overexpression of KIM-1 in the carcinoma cells *in vitro* correlates with the greater activity of their proliferation and increased IL-6 production. The induction of IL-6 expression directly depended on the intensity of KIM-1 shedding from the tumor cell surfaces. The authors believe that a free extracellular domain KIM-1 is capable of activating the signaling axis KIM-1/IL-6/STAT-3 via the paracrine or autocrine mechanism.

Irrespective of the KIM-1 expression level in the tumor cells, its increased synthesis is observed in the epithelium of the renal proximal tubular cells in the unchanged kidney tissue surrounding malignant tumor. It may be a consequence of the tissue compression and ischemia caused by progressive growth of the neoplasm [128]. However, Cuadros et al. [130] came to a conclusion that in the clear cell renal carcinoma, the elevated KIM-1 expression in the morphologically normal adjacent tissue does not depend on the tumor growth and reflects most likely individual predisposition to RCC development.

As have already been mentioned, increase of KIM-1 expression in chronic hypoxia can maintain low-grade inflammation, and the shed extracellular KIM-1 domain is able to interact with the tumor stroma components, i.e. endothelial cells, tumor-associated myeloid cells, and lymphocytes. Interstitial inflammation serves as a source of growth factors and cytokines stimulating proliferation of cancer cells and neoangiogenesis and also attracts lymphoid and myeloid suppressor cells leading to the suppression of the innate and acquired immunity, inhibition of cytotoxic lymphocyte activity [137]. Thus, aberrant KIM-1 expression in RCC cells and/or in the renal tubular epithelium in the tissue surrounding the tumor can make a definite contribution to the formation of tolerant microenvironment and to the escape of cancer cells from immune surveillance.

Increased KIM-1 expression in the renal tissue in RCC is accompanied by its greater content in urine and blood plasma [128, 130, 131, 138–140]. Concentration of uKIM-1 in RCC patients exceeds statistically significantly that in healthy individuals, and elevation of the uKIM-1 levels correlates with the disease stage, tumor size, and tumor grade [130, 138, 140]. After nephrectomy, uKIM-1 concentration reduces and approximates normal values [131, 138, 140], which points to the tumor as a direct source of KIM-1 in the patients’ urine.

Zhang et al. [131] note a direct correlation between uKIM-1 and the level of KIM-1 expression in the RCC cells. According to other studies [128, 141], increased uKIM-1 levels are also observed in chromophobe RCC which is not typically express KIM-1. The source of the elevated uKIM-1 in these cases is likely to be the altered renal parenchyma rather than the cancer cells [128].

In patients with clear cell or papillary RCC, statistically significant increase of KIM-1 concentration in blood plasma is observed relative to healthy donors. pKIM-1 levels in these patients correlate with the cancer stage while in patients with chromophobe RCC, pKIM-1 does not practically differ from that in healthy individuals [142]. According to the data of the multicenter prospective cohort study EPIC (European Prospective Investigation into Cancer and Nutrition), the increase of the pKIM-1 level may serve as a predictor of the RCC risk: in the group of individuals with the revealed RCC during diagnostic monitoring, an average pKIM-1 level in the previous years was 2.5 times higher than in healthy donors [143]. The authors believe that increased pKIM-1 without specific symptoms increases 63 times the probability of malignant neoplasm development in the kidneys during the following 5 years.

## KIM-1 in malignant tumors of extra-renal localization

KIM-1 overexpression in the cells of clear cell and papillary RCC has for a long time been considered as a unique feature of kidney tumors. Performing immunohistochemical staining of 484 specimens of tumors of extra-renal origin using monoclonal antibody AKG7, Han et al. [128] did not reveal KIM-1 expression in most cases. These data are quite in line with the assessment of KIM-1 mRNA expression: the content of *HAVcr-1* transcripts in the renal malignant tissues is 10 times higher than that in malignant tumors of other localization [7].

Later, KIM-1 expression was described in clear cell carcinoma of the ovary (93.8%) and endometrium (33.3%), in colon carcinoma (12.5%) [129], germ cell tumors (50%) [144], as well as in the cells of primary lymphoma of the central nervous system (54%) [145].

According to the data obtained from the Human Protein Atlas database [7], a high intensity staining the tumor cells with anti-HAVcr-1 polyclonal antibodies was detected in breast and stomach cancer, a weak or moderate intensity was revealed in colon cancer, pancreas, non-small cell lung cancer, and ovarian carcinoma. Disagreement in the assessment of the HAVcr-1/KIM-1/TIM-1 expression in cancer tissues may be explained by different sensitivity of the immunochemical methods or unique epitope specificity of AKG7 antibody [59].

Data on clinical significance of increased KIM-1 expression in the tumors of extra-renal localization are ambiguous. Thus, in stomach cancer, an elevated expression of KIM-1 mRNA is associated with unfavorable prognosis and low sensitivity to chemotherapy [146]. *HAVcr-1* knockdown reduces the activity of proliferation and colony formation, migration, and invasion of gastric cancer cells *in vitro* [147]. In non-small cell lung cancer, increased KIM-1 expression at the level of protein and mRNA is also associated with a worse patients’ survival [148]. Inactivation of KIM-1 in А549 and SK-MES-1 lung cancer cells suppresses proliferation, migration activity, and invasion and is also accompanied by the rise of the level of tumor suppressor protein PTEN and inhibition of prooncogenic PI3K/Akt signaling pathway [148]. At the same time, overexpression of KIM-1 mRNA in the colon cancer tissue is associated, on the contrary, with a longer recurrence-free survival of patients [149]. Transfection of the colon cancer cells with *HAVcr-1* gene does not influence the growth rate and cell motility *in vitro* but reduces their invasion ability [149].

No correlation has been found between the level of KIM-1 expression in the cancer cells and clinical and morphological characteristics of the mentioned malignant diseases, which indicates independent prognostic significance of this indicator.

## Conclusion

The accumulated store of knowledge forms today the notion on HAVcr-1/KIM-1/TIM-1 as a multifunctional evolutionally conservative molecule resembling two-faced Janus. On the one hand, KIM-1 is involved in homeostasis maintenance participating in the regulation of immune reactions and supporting functional state of renal tubular epithelium in case of ischemic and toxic stress. On the other hand, KIM-1 is used by highly pathogenic viruses for penetration into the cells. Its long expression in the proximal tubular cells in kidneys promotes the development of fibrotic changes while increased expression in the cells of RCC and some other malignant tumors may be the factor provoking tumor progression. Molecular mechanisms determining the role of KIM-1 in normal and pathological conditions and the meanings of these processes still are waiting for deep exploration. Nevertheless, predicting properties of KIM-1 as an urinary and serologic marker in some kinds of acute and chronic kidney injury, renal cell carcinoma, cardiovascular diseases may already be used in the current clinical practice.
